# Natural selection on traits and trait plasticity in *Arabidopsis thaliana* varies across competitive environments

**DOI:** 10.1038/s41598-020-77444-w

**Published:** 2020-12-10

**Authors:** Kattia Palacio-Lopez, Christian M. King, Jonathan Bloomberg, Stephen M. Hovick

**Affiliations:** 1 Department of Evolution, Ecology and Organismal Biology, The Ohio State University, Columbus, OH USA; 2 London School of Hygiene and Tropical Medicine, London, UK

**Keywords:** Ecology, Evolution, Plant sciences

## Abstract

Interspecific competition reduces resource availability and can affect evolution. We quantified multivariate selection in the presence and absence of strong interspecific competition using a greenhouse experiment with 35 natural accessions of *Arabidopsis thaliana*. We assessed selection on nine traits representing plant phenology, growth, and architecture, as well as their plasticities*.* Competition reduced biomass and fitness by over 98%, and plastic responses to competition varied by genotype (significant G × E) for all traits except specific leaf area (SLA). Competitive treatments altered selection on flowering phenology and plant architecture, with significant selection on all phenology traits and most architecture traits under competition-present conditions but little indication that selection occurred in the absence of competitors. Plasticity affected fitness only in competition-present conditions, where plasticity in flowering time and early internode lengths was adaptive. The competitive environment caused changes in the trait correlation structure and surprisingly reduced phenotypic integration, which helped explain some of the observed selection patterns. Despite this overall shift in the trait correlation matrix, genotypes with delayed flowering had lower SLA (thicker, tougher leaves) regardless of the competitive environment, a pattern we have not seen previously reported in the literature. Overall, our study highlights multiple ways in which interspecific competition can alter selective regimes, contributing to our understanding of variability in selection processes over space and time.

## Introduction

Plants regularly compete for key resources with their neighbors, which negatively impact plant fitness through reduced access to those resources^1^. Competition can also promote the maintenance of genetic diversity within plant populations^2,3^, and, where competitor effects vary from place to place, evolutionary change in response to competition can lead to phenotypic divergence among populations^4–7^. Such divergence can result from variable selection on traits that enhance resource capture in diverse populations or under specific competitive environments^8–10^.

The role of selection on phenotypic traits in response to competition has mostly focused on traits related to vegetative growth strategy or reproductive timing^11^. For example, early stem elongation is often selected for in the presence of competitors to maximize light interception, indicating that timing of vegetative growth is essential to overall success^5,12,13^. With respect to reproductive timing, the age at which a plant transitions from vegetative growth only to flowering has a large impact on fitness, particularly under limiting environmental conditions^14–18^. In the presence of competition, selection often favors earlier flowering time, reflecting an increased allocation of resources to reproduction in early life-history stages^4,19–22^.

Although studies like these demonstrate selection on individual traits in the presence of competition, it is also important to consider selection across multivariate trait syndromes in these conditions. Few studies have examined selection on multiple traits across several trait categories in plants exposed to competition (but see^19,23^). Trait category in this case refers to broad suites of plant traits grouped according to their influence on (for example) phenology, physiology, and morphology^11^. By investigating how phenotypic traits vary in response to competition as well as how those trait values co-vary among themselves and with relative fitness^24^, it is possible to assess how selection influences multivariate phenotypes in variable competitive environments. Such a holistic approach is valuable because it helps to account for constraints imposed by inter-trait correlations and how those correlations may themselves change across competitive regimes^25^. Because the array of phenotypes in a population under a particular environmental condition reflects a diversity of traits and how those traits co-vary with one another (phenotypic integration), the overall genetic architecture underlying trait correlations will often impose significant constraints on trait evolution^26,27^. Understanding how such constraints relate to environment-specific trait correlations can thus yield valuable insights about how responses to selection vary over time and space^28,29^. For example, in some cases selection could indirectly lead to non-adaptive values of individual traits as a result of genetic correlations^30^.

Selection may also favor plants that maintain fitness in competition with neighbors via phenotypic plasticity, the ability of a single genotype to produce different phenotypes in response to variable environments^31^. Plastic responses have been identified as a mechanism by which organisms can reduce the negative effects of limiting growing conditions^8,31–34^, such as competition^19,23,35^. Competitive environments provide a key context in which the ability to be phenotypically plastic should theoretically be favored in plants, because competitor presence changes environmental cues in a reliable way (e.g., by reducing the ratio of red to far red light^5,12^) and because competitive environments vary over time and space^36^. Plastic responses can enhance performance (adaptive plasticity) under widely varying conditions and therefore be selected^35,37^. Alternatively, plastic responses can constrain fitness (maladaptive plasticity) and be selected only indirectly due to genetic correlations^34,38^. Selection for plasticity can also be inhibited due to various costs of and constraints to plastic responses^33,39–42^, although recent work has argued that for the majority of species those costs should be minimal^43^. Measurement of plasticity costs is limited by the lack of independence between trait and trait plasticity values, as well as by environment-specific fitness implications for both^44^. However, approaches that allow for inferences regarding whether a plastic response is beneficial or not across different environments are still important for understanding the evolution and maintenance of plasticity^37,45^.

A common quantitative approach for estimating the strength of natural selection on a diversity of phenotypes is genotypic selection analysis (GSA)^46^. GSA assumes that all genetic variation for traits is additive. By using genotype mean values, this approach reflects genetic correlations and can account for the omission of genetically non-variable traits that covary with fitness. In addition, GSA can provide a less biased estimate of the strength of selection when environmental conditions covary with fitness and phenotypic traits^46^. GSA uses a multiple regression framework to determine the relationship between a trait and relative fitness, known as a selection gradient (β, the regression coefficient)^24^. Such an approach is well-suited for estimating direct linear or nonlinear selection on individual traits, accounting for trait covariances with all other traits included in the analysis. Estimates of direct selection can also help clarify instances of indirect selection, where a trait is not selected upon directly but instead covaries with fitness primarily because of its phenotypic correlations with one or more traits that are directly selected upon^46^. Thus, inferences regarding indirect selection are based on instances where total selection is significant, based on selection differentials (S, the covariance between a trait and relative fitness), but direct selection, as indicated by significant selection gradients, is not.

Selection analyses conducted across differing environmental conditions have been used to highlight substantial variation in the magnitude and directionality of natural selection on key traits (e.g. in^47,48^). Generalizable insights from reviews of such studies have been limited, including no support for the prediction that selection magnitudes should generally increase under stressful conditions^49,50^. Yet, competitive environments appear to represent an exception, with consistent reports of stronger selection when neighbor densities are increased^50^. In environments with size-dependent competitive hierarchies, individuals with traits that give them even a slight fitness advantage may realize increasing relative fitness gains as neighbor densities increase^49^. Conversely, when densities decrease, individuals are exposed to more benign environments in which resource limitation is relaxed, leading to weaker selection compared to highly competitive environments. By this reasoning, competition should increase selection strength on relevant traits, perhaps leading to adaptation in populations^51^. Yet in reality, the net observed evolutionary change in response to any stress will necessarily reflect a balance between standing trait variation and the strength of selection^52–54^.

We were interested in quantifying how natural selection on a set of key phenotypic traits and their plasticity would vary across contrasting competitive environments. Although some responses to competition are species-specific^55^, many are not^56–58^; we therefore consider selective responses to competition to be largely generalizable regardless of competitor identity. In this study we were interested primarily in these general strategies that might hold across species in their response to interspecific competition. Under greenhouse conditions, we conducted an experiment to evaluate phenotypic responses of the model annual plant *Arabidopsis thaliana* (hereafter *Arabidopsis*) growing either alone or in competition with annual rye *Lolium multiflorum* to test the following hypotheses: (1) Both total and direct selection will be greater in absolute value in the presence of competition, (2) Phenotypic plasticity will be selected for, especially in highly competitive conditions, and (3) Differing selection in competitive versus non-competitive conditions will reflect in part an environment-dependent shift in the underlying trait correlations.

## Results

### General effects of competition on plant traits and performance

**Figure 1:**
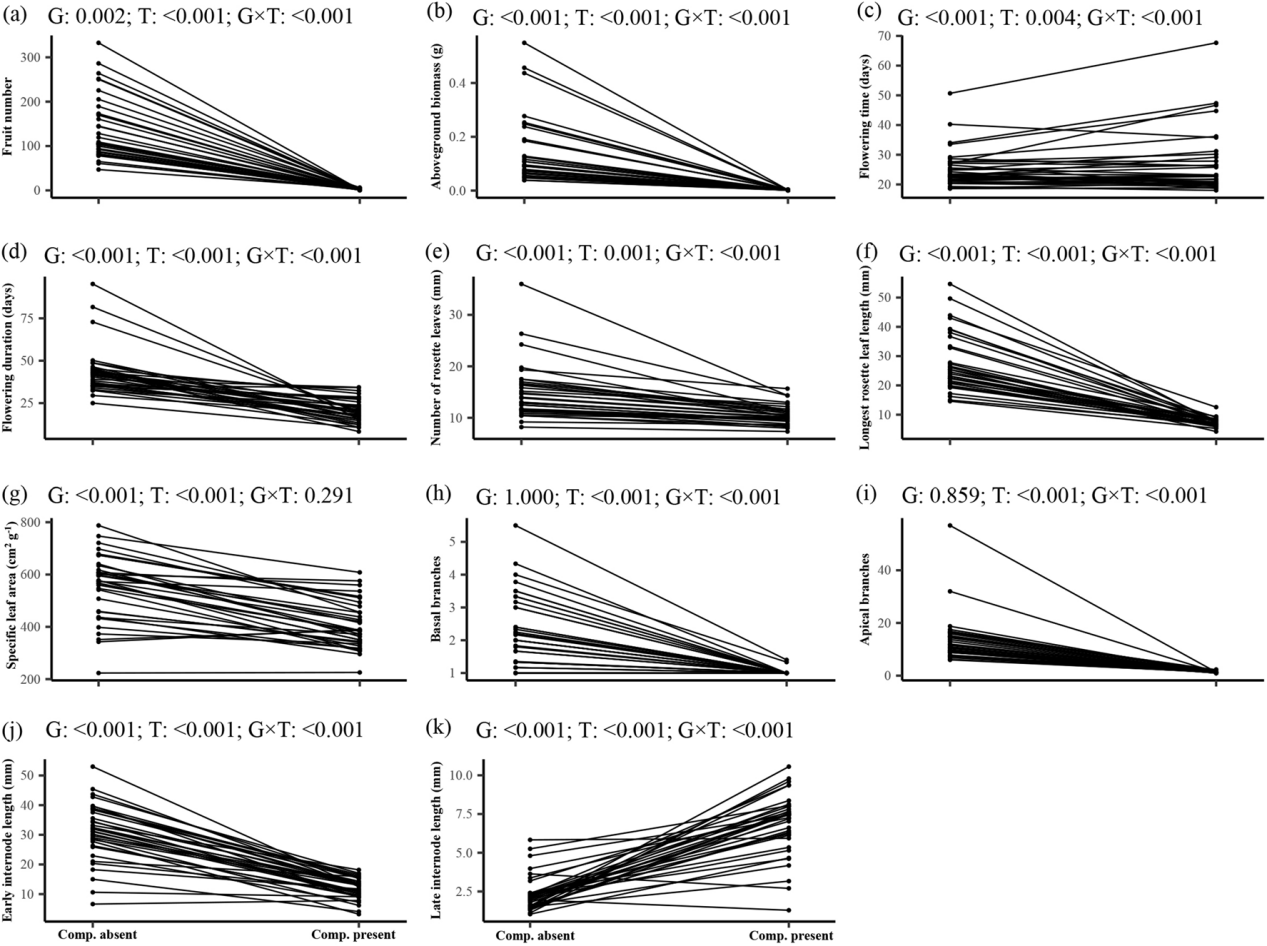
Reaction norms for performance and trait variation in response to competition treatments across thirty-five *Arabidopsis* genotypes. Each point represents a genotype mean value in either competition-absent or competition-present conditions. *P* values from linear mixed models testing the effect of genotype (G), treatment (T) and their interaction (G × T) are shown.

*Arabidopsis* growing in the presence of *L. multiflorum* showed a 98.6% reduction in size and 97.9% reduction in fecundity compared to plants growing alone, (Fig. 1a,b; *P* < 0.001). Competition also led to significant changes in all other measured traits (Fig. 1c–k; all *P* ≤ 0.005), usually reflecting decreases in trait values. The two exceptions were in flowering time (Fig. 1c) and late internode lengths (Fig. 1k), which increased with competition. Significant genotype × competition treatment interactions for all measured traits except specific leaf area (SLA) indicate that plasticity in response to competition varied among genotypes (Fig. 1; all interaction *P* < 0.001 except for SLA). This differential plasticity mostly reflects variability in the strength of plastic responses (the reaction norm slopes), but for flowering time and late internode lengths our genotypes also varied in the direction of trait change, experiencing either increases or decreases in response to competition (Fig. 1c,k). No single genotype (or suite of genotypes) consistently outperformed the others across competitive environments for the traits and trait plasticities we recorded (Table S4). The presence of competitors significantly reduced total variability in trait expression for most traits (*P* ≤ 0.05, Levene’s test), but especially basal branch number showed a drastic reduction, (for which 33 of 35 genotypes had no basal branches in competition; Fig. 1h, Table S4). Flowering time and late internode lengths had increased variability in competitive conditions (*P* ≤ 0.05, Levene’s test). Variance in flowering duration and SLA did not differ based on competitive conditions.

### Selection on phenology traits

**Figure 2:**
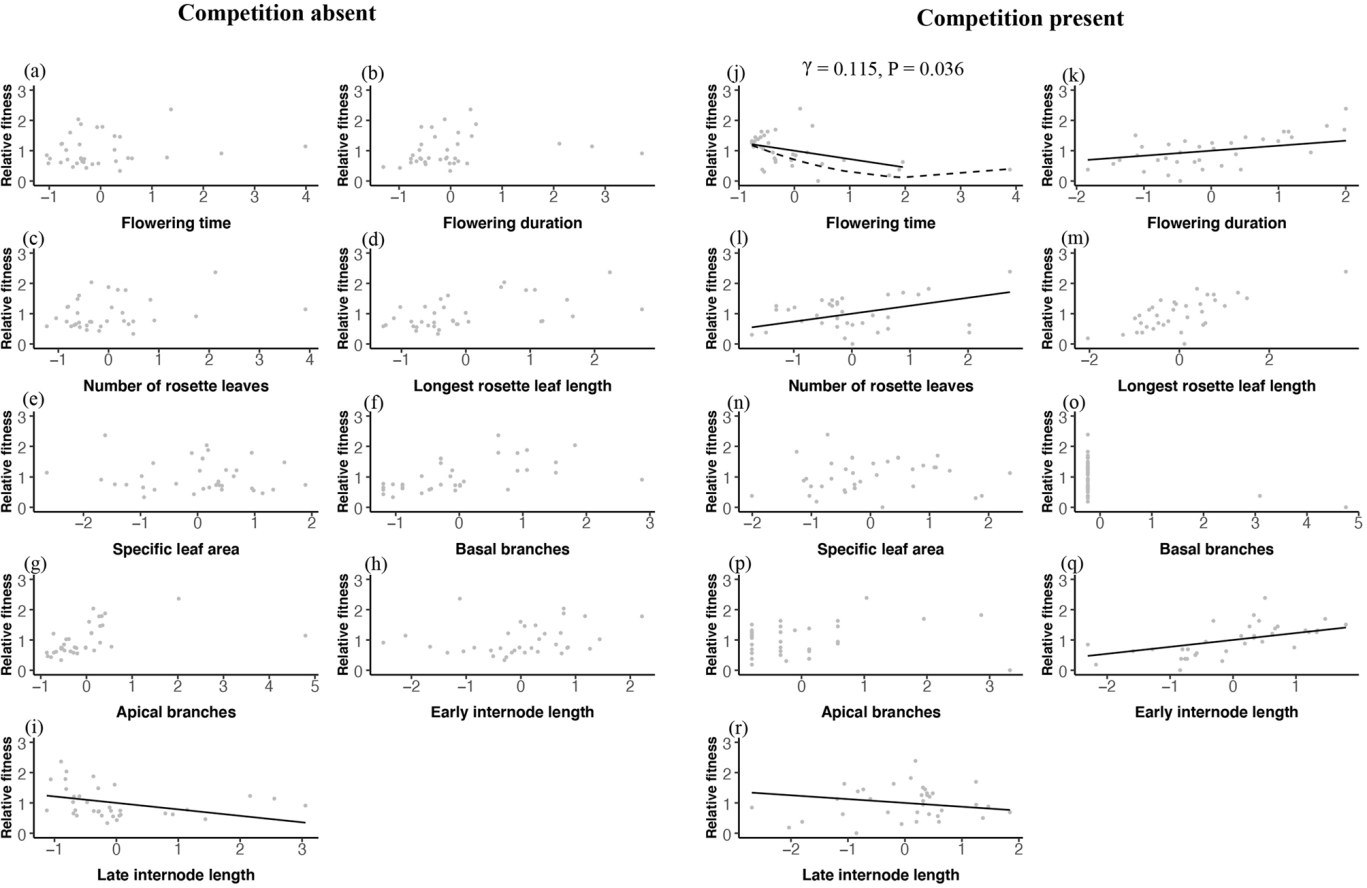
Relationships between relative fitness (fruit number) and standardized trait values from phenotypic selection models in competition-absent (**a**–**i**) and competition-present (**j**–**r**) conditions. Solid lines represent significant selection gradients (*β*), based on model parameter estimates in Table 1. Dashed lines represent significant nonlinear relationships, based on parameter estimates from the quadratic phenotypic selection model (γ and *P* values shown for all cases with *P* < 0.05).

Selection on *Arabidopsis* phenology traits contrasted sharply for plants grown under varying competitive conditions, based on preliminary analysis with data from both competitive treatments in which trait × treatment interactions were significant for four out of ten traits. We therefore report results from GSA conducted in each competition treatment separately (Table 1). Earlier flowering was selected only under competition-present conditions (*β* = − 0.276), including significant nonlinear selection (γ = 0.115) that indicated a sharp decrease in relative fitness with even a slight delay in flowering time (Fig. 2j). In the presence of competitors, selection also favored extended flowering durations (*β* = 0.165; Fig. 2k) and the production of more rosette leaves prior to flowering initiation (*β* = 0.262; Fig. 2l), despite the fact that genotypes with more rosette leaves at bolting tended to flower later (r = 0.571, Table 3). These results contrasted with a lack of selection on phenology traits in the absence of competition (Table 1, Fig. 2a–c).

**Table 1:** Results of genotypic selection analysis showing selection gradients (β) with standard error values (SE) and selection differentials (S) for each competition treatment.

	Competition absent					Competition present				
	*β*	SE	*P*	S	*P*	*β*	SE	*P*	S	*P*
**Phenology traits**										
Flowering time	− 0.192	0.211	0.373	0.150	0.389	− **0.276**	**0.069**	**0.001**	− **0.462**	**0.005**
Flowering duration	− 0.098	0.123	0.436	0.202	0.245	**0.165**	**0.057**	**0.007**	**0.659**	**< 0.001**
Number of rosette leaves	− 0.288	0.256	0.271	0.274	0.111	**0.262**	**0.098**	**0.013**	0.273	0.112
**Growth traits**										
Longest rosette leaf length	0.203	0.123	0.112	**0.536**	**0.001**	0.110	0.079	0.177	**0.718**	**< 0.001**
Specific leaf area	− 0.033	0.116	0.782	− 0.110	0.530	− 0.018	0.056	0.742	0.094	0.590
**Architectural traits**										
Basal branches	0.136	0.110	0.227	**0.580**	** < 0.001**					
Apical branches	*0.198*	*0.105*	*0.072*	**0.450**	**0.007**	− *0.094*	*0.048*	*0.064*	0.154	0.377
Early internode lengths	− 0.087	0.079	0.282	0.237	0.170	**0.228**	**0.072**	**0.004**	**0.646**	**< 0.001**
Late internode lengths	− **0.213**	**0.080**	**0.013**	− 0.252	0.144	− **0.126**	**0.046**	**0.012**	0.130	0.456

Significant terms (*P* < 0.05) are shown in bold and marginally significant terms (0.05 < *P* < 0.10) in italics.

Plasticity in flowering time showed an adaptive response for plants in the competition-present treatment (*β* = 0.254; Table 2) but not in the competition-absent treatment; these results may reflect the fact that our most plastic genotypes were also the earliest flowering, but that this correlation held only in competitive conditions (competition present: r = − 0.740, *P* < 0.001; competition absent: r = − 0.278, *P* > 0.05; Table S2). Plasticity in flowering duration and the number of rosette leaves co-varied negatively with fitness in the presence of competition, but these effects were significant only in the simple model without correlated traits included (Table 2).

**Table 2:** Results of selection analyses to estimate the extent to which plasticity is adaptive or maladaptive.

	Competition absent				Competition present			
	*β*_full_	*P*_full_	*β*_simple_	*P*_simple_	*β*_full_	*P*_full_	*β*_simple_	*P*_simple_
**Phenology traits**								
PP Flowering time	− 0.093	0.337	− 0.063	0.313	**0.254**	**0.023**	− 0.079	0.421
PP Flowering duration	− 0.132	0.134	− 0.011	0.888	− 0.053	0.665	− **0.365**	**0.007**
PP Number of rosette leaves	0.014	0.936	− 0.070	0.559	− 0.183	0.185	− **0.364**	**< 0.001**
**Growth traits**								
PP Longest rosette leaf length	0.063	0.715	0.013	0.914	− 0.008	0.925	− **0.223**	**< 0.001**
PP Specific leaf area	0.110	0.163	*0.151*	*0.063*	0.028	0.741	**0.276**	**< 0.001**
**Architectural traits**								
PP Basal branches	− 0.062	0.708	**0.335**	**0.018**				
PP Apical branches	0.106	0.219	**0.247**	**0.005**	*0.123*	*0.098*	− **0.234**	**0.010**
PP Early internode lengths	0.044	0.693	**0.171**	**0.048**	**0.265**	**0.009**	**0.174**	**0.010**
PP Late internode lengths	− 0.108	0.339	− 0.012	0.891	*0.151*	*0.063*	− 0.114	0.295

Negative values of the coefficient for plasticity (*β*) indicate maladaptive plasticity, while positive values indicate adaptiveness. Significant terms (*P* < 0.05) are shown in bold and marginally significant terms (0.05 < *P* < 0.10) in italics.

### Selection on growth traits

Growth traits and their plasticities were not a target of linear or nonlinear selection in either competition treatment based on selection gradients (Tables 1, 2, Fig. 2d,e,m,n). Rosette leaf length at flowering was positively correlated with fitness under both competitive environments to a similar extent, according to selection differentials (S = 0.536 in competition-absent and S = 0.718 in competition-present conditions; Table 1). Based on simple models only, plasticity in rosette leaf length was maladaptive in competition-present conditions and plasticity in SLA was selectively favored in both treatments (Table 2). Rosette leaf length plasticity is highly correlated with flowering time (r = 0.663, *P* < 0.001) and its plasticity (r = − 0.416, *P* < 0.05), which could explain why it was not a significant predictor of relative fitness when those traits were accounted for, despite co-varying with fitness itself. A similar shift for SLA plasticity may reflect its high correlation with plasticity in early internode lengths (r = 0.588, *P* < 0.001).

### Selection on architectural traits

Selection on architectural traits contrasted in competition-present versus competition-absent treatments, favoring longer early internodes in the competition-present treatment (*β* = 0.228; Fig. 2q) but not in the competition-absent treatment (Fig. 2h). Shorter late internodes were selected in both competition-present (*β* = − 0.126) and competition-absent conditions (*β* = − 0.213; Table 1; Fig. 2i,r). Apical and basal branches were not a target of linear selection in either competition treatment based on selection gradients (Fig. 2f,g,o,p; Table 1). We found no indication of nonlinear selection on architectural traits.

Plastic responses in architectural traits were adaptive under competition-present conditions, including selection for plasticity in early internode lengths (*β* = 0.265; Table 2). Plasticity on apical branch numbers and late internode lengths were marginally significant in competition (Table 2).

### Correlations between phenotypes and plasticity

**Figure 3:**
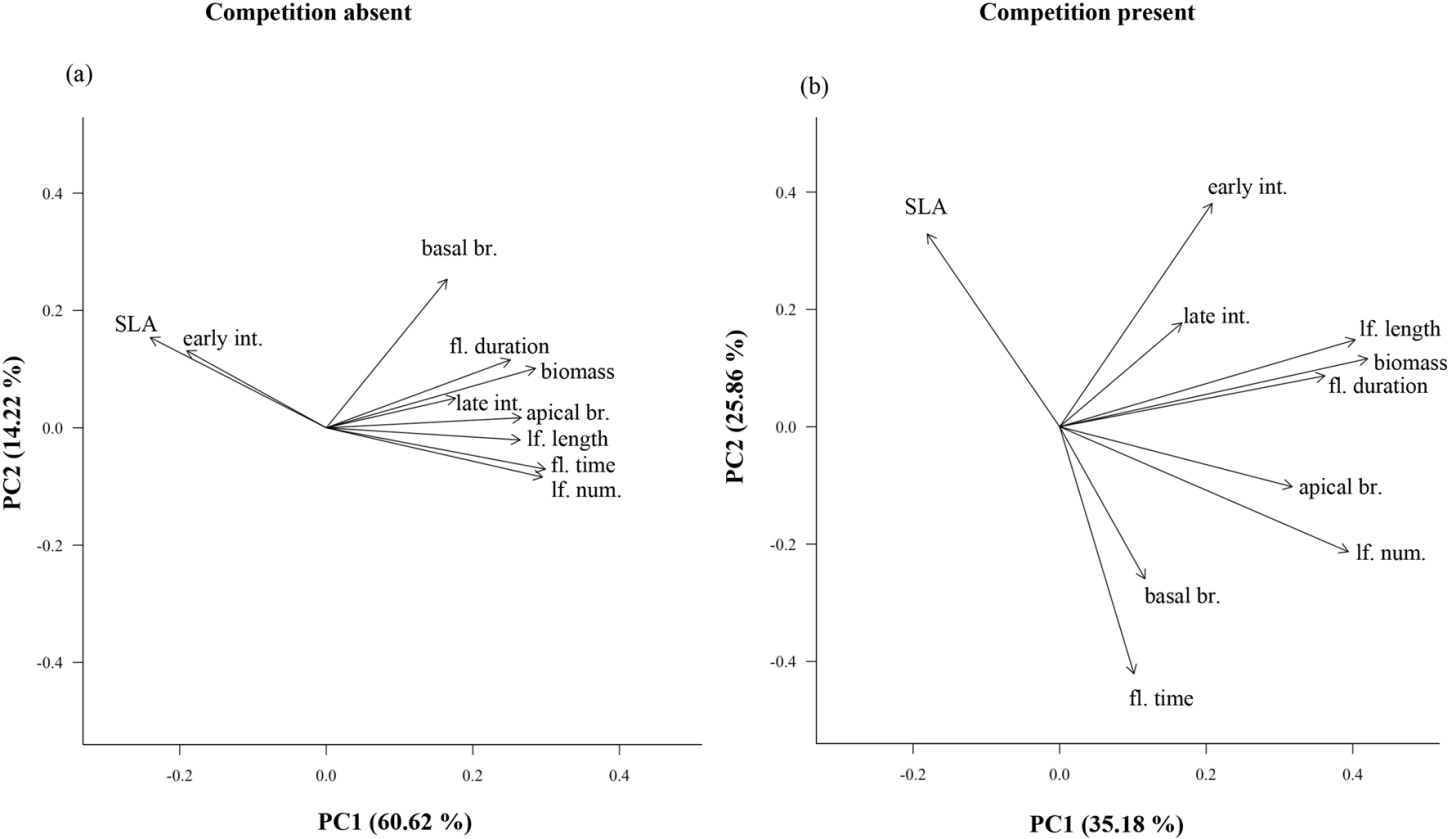
Principal components analysis output, showing relationships among our nine focal traits plus biomass for 35 genotypes of *Arabidopsis thaliana* grown without competition (**a**) and with competition (**b**). Each arrow represents the loading values for a given trait on principal components axis 1 (PC1) and axis 2 (PC2). Abbreviations are as follows: fl. time, flowering time; fl. duration, flowering duration; lf. num., number of rosette leaves; lf. length, longest rosette leaf length; SLA, specific leaf area; biomass, aboveground biomass; basal br., number of basal branches; apical br., number of apical branches; early int., early internode length; late int., late internode length.

Based on treatment-specific PCA with all measured trait values, *Arabidopsis*’ trait correlation matrix shifted in response to the competitive environment, particularly with respect to flowering time, SLA and early internode lengths (Fig. 3; Table 3; see Table S3 for PCA loadings). In both treatments, the first PC axis primarily reflects variation in plant size and most of our phenology traits, with high loading values for aboveground biomass, flowering duration, rosette leaf number and size, and apical branch numbers (Fig. 3, Table S3). Under competition-absent conditions, the first axis also corresponds to variation in flowering time and SLA (Fig. 3, Table S3). The second PC axis primarily reflects variation in number of basal branches under competition-absent conditions, but flowering time, SLA and early internode lengths under competition-present conditions (Fig. 3, Table S3).

**Table 3:**
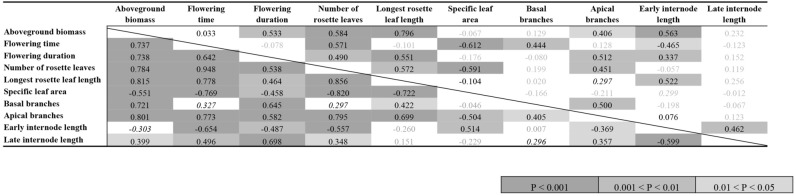
Correlation matrix for traits in competition-absent (below diagonal) and competition-present (above diagonal) conditions.

Significant terms (*P* < 0.05) are shown in bold with increasingly darker shading indicating trait correlations with lower *P* values (in three categories: *P* < 0.001; 0.001 < *P* < 0.01; and 0.01 < *P* < 0.05); marginally significant correlations (0.05 < *P* < 0.10) are shown in italics.

Larger plants tended to flower longer with a greater number of leaves regardless of treatment, but correlations between flowering time and plant size were treatment specific (Table 3). Plant size was not correlated with flowering time under competition-present conditions (r = 0.033, *P* > 0.1), but larger plants did flower later under competition-absent treatments (r = 0.737; *P* < 0.001; Table 3, Fig. 3). These treatment-specific patterns of flowering time variation corresponded with relatively consistent variation in SLA, such that delayed flowering was associated with thicker, tougher leaves (low SLA) in both competition-present (r = − 0.612; *P* = 0.001) and competition-absent conditions (r = − 0.769; *P* < 0.001). In the PCA, we thus find a flowering time—SLA gradient, which is largely indicative of plant size and phenology variation in the absence of competition but is mostly orthogonal to that axis when competition is present (Fig. 3). Although SLA is correlated with the majority of our nine other focal traits in competition-absent conditions (7 correlations significant, only basal branches and late internode length were not correlated with SLA), SLA is only correlated with 3 of 9 (flowering time, number of rosette leaves and marginally with early internode length) in competition-present conditions (Table 3). In fact, across all traits more pairs were significantly correlated in competition-absent (36 of 45) than in competition-present conditions (19 of 45). This pattern of reduced phenotypic integration in competition-present compared to competition-absent conditions also held when we included correlations with trait plasticity values (Table S2).

## Discussion

We characterized multivariate phenotypes and fitness for 35 accessions of *Arabidopsis* in order to test how selection varies in response to interspecific competition. Although fitness in *Arabidopsis* was reduced in the presence of competitors, we observed substantial variation among accessions in responses of individual traits. We also found environment-dependent patterns of natural selection based on competitive conditions that play out across our phenology, growth and architectural traits. Such variability may reflect a soft selection model, in which density of neighbors (or intraspecific density) regulates phenotypic responses locally^59,60^. Although we did not find overwhelming evidence for selection on phenotypic plasticity, we did observe adaptive plasticity in flowering time and early internode lengths in the presence of strong competition. Given the fundamental role played by flowering time and architectural traits such as early internode length in response to competition, these findings are likely relevant beyond the context of this individual study. Lastly, we document an environment-dependent shift in the overall trait correlation matrix, including the surprising finding that phenotypic integration was reduced under more stressful growing conditions and that changes in correlations between SLA and flowering time versus all other traits were fundamental to how those correlation matrices varied across competitive environments.

### Variable selection on plant traits in contrasting competitive environments

#### Flowering phenology

Our results highlight important similarities and differences in selection on phenology depending on the presence of competitors. Earlier flowering in *Arabidopsis* was selected under competition-present but not competition-absent conditions. Selection favoring earlier flowering (negative *β*) has been also reported in recent studies^61,62^. In annual plants, the transition from vegetative growth to reproductive maturity is strongly influenced by resource availability^63–65^ and biotic interactions^14,66^ thus flowering earlier can be favored when doing so reduces the negative effects of factors such as competition and seed predation. Our results show the role of high-density environment as a strong selective agent, contrasting with the observation that selection on flowering time was nonsignificant under benign, competition-absent conditions (as in Weinig et al^19^).

Flowering time is also a key life-history trait in annual plants like *Arabidopsis* because it directly influences biomass allocation tradeoffs and thus fitness^14,63^. Our results indicate a close relationship between growth and phenology traits as they relate to fitness, with bigger plants gaining a fitness advantage in both competitive environments. For our plants grown without competitors, size at bolting and flowering time were positively correlated (i.e., late flowering genotypes grew larger before flowering), consistent with expectations and with previous work on *Arabidopsis*^67^*.* However, growing under stressful conditions due to competition or other factors adds a level of complication in terms of balancing reproductive phenology and growth traits^68^. Despite the fitness benefits of growing larger prior to becoming reproductive, under our competition-present conditions selection still strongly favored early flowering.

We also found a contrasting adaptive strategy across competition treatments regarding the number of rosette leaves, which is a phenological indicator of the developmental stage at which *Arabidopsis* shifts from being vegetative to reproductive. Under competition present conditions, selection favors plants that are more developmentally advanced at flowering (positive *β* and S; see Table 1). The range of leaf number values observed across competitive environments overlaps for most individuals (Fig. 1e), which may indicate some degree of developmental canalization in the accessions we used^69^. However, estimates of direct selection indicate that growing more leaves before flowering is selected under competition-present conditions, whereas in the absence of competition this trait is not selected. This discrepancy reflects a strong correlation between flowering time and the number of rosette leaves when *Arabidopsis* is grown alone (r = 0.948) that weakens substantially in the presence of competition (r = 0.571; see also Fig. 3). Flowering time responses represent the integration of multiple cues from the external environment along with various intrinsic factors^70^. But, understanding the nature of trade-offs among phenology traits is also necessary to clarify the drivers of reproductive success, especially in light of resource acquisition thresholds for flowering that may present challenges in resource limited conditions^67^. It is not entirely clear why correlations with flowering time would be so much weaker in the presence of competition, but our results highlight the overarching importance of flowering time, and the correlations and trade-offs between flowering time and other phenology traits, for fecundity selection across environments.

#### Architectural traits

Architectural traits such as the number of basal and apical branches have previously been shown to influence fitness in *Arabidopsis*^4,19,23,71–73^. A close connection between fecundity and branch numbers in *Arabidopsis* is expected given that increasing the number of branches leads to more flower-bearing meristems^73^. In the absence of competition, we observed substantial variation in branch numbers among our accessions but only weak indicators of selection for greater branching (significant total selection, S, but not direct selection, *β*). This pattern could result from our inclusion of biomass in the full GSA model, if the effect of branch number makes little difference beyond the influence of biomass on relative fitness. Perhaps more surprising, and contrary to patterns in the absence of competition, is that genotypes growing with competitors almost entirely failed to produce basal branches, with all lateral growth occurring from the main stem. Yet, even the number of apical branches was not selected under competition-present conditions. We believe this is the result of a drastic reduction in trait variation in response to harsh growing conditions. Plants produced between 6 to 57 apical branches under competition absent conditions but only 1 to 3 under competition present conditions.

For architectural traits related to stem elongation, we found an interesting developmental difference in selection under competition-present conditions: elongation in early developing internodes is selected for, but elongation in later developing internodes is selected against. Increasing stem elongation in early developmental stages when neighbors are present is a common response that can increase resource capture and thus lifetime fitness in *Arabidopsis*^23^ and other annual plant species such as *Impatiens capensis*^12,74,75^. Because increased stem elongation in late developmental stages is selected against in both of our treatment conditions, it seems that stem elongation late in development may be universally maladaptive. Selection against late internode elongation has been reported before but only under no-competition conditions^19^.

### Selection on trait plasticity in contrasting competitive environments

We show that plastic responses are more selectively important under competition-present than competition-absent conditions, although this is not evident across all measured traits. The standard statistical approach for determining the role of selection on trait plasticity^17,19,62,76–78^, which we also used here, has been revised on multiple occasions highlighting its complexities and limitations^38,44,45,79^. One of the most commonly discussed limitations of this approach is the inclusion of both a trait and its plasticity in the same model despite the non-independence of these values^44^. Because of this limitation, our findings about selection on plasticity must be interpreted with some degree of skepticism, rather than treating our results as authoritative. Alternative methods for making inferences about selection on plasticity that overcome these limitations would be desirable, but to our knowledge, such methods do not yet exist.

There are three basic conditions for the evolution of plasticity to occur: genetic variation in plasticity, correlations between plasticity and fitness, and heterogeneous conditions leading to different phenotypes being favored in different times or places^33,39,80,81^. Our results support all three conditions in *Arabidopsis*. The first, genetic variation in plasticity, was ubiquitous among our *Arabidopsis* genotypes, as indicated by significant genotype × environment (G × E) interactions^33^ for all traits except SLA.

The second condition, a significant correlation between plasticity and fitness^80^, is supported in our dataset for plasticity in flowering time and early internode lengths under competition-present conditions. The importance of early flowering and its ability to fluctuate depends on the environment and appears to be fundamental for *Arabidopsis* success under competition-present conditions, as discussed previously. Selection for early internode length plasticity has been reported previously in experiments with *Arabidopsis* and *Impatiens*^12,19^*.* In our study, selection favored longer early internodes in the presence of competition (see above), and its ability to change in response to different competitive environments. Interestingly, we found no other indication that plasticity in any other trait enhanced fitness. Although this result is consistent with research suggesting that selection on plasticity is uncommon^44,82^, it contrasts somewhat with the overall findings of Weinig et al.^19^ who found evidence for adaptive plasticity in two out of six measured traits (basal branches and apical inflorescence elongation) under competition conditions in *Arabidopsis* recombinant inbred lines. Although our studies overlap to some extent, a distinct difference is that we used natural accessions of *Arabidopsis.* The plant material used by Weinig et al.^19^ had thus not yet been exposed to natural selection to filter out genotypes that may have been responsible for an artificially strong signal for the relationship between plasticity and fitness. In addition, we are reporting phenotypic responses to interspecific competition rather than intraspecific competition^19^. Because the spatial and temporal variation generated by plastic responses reflects in part the density and identity of the interacting organisms, plasticity can have broad implications for the evolutionary outcome of ecological interactions^83,84^. *L. multiflorum* is a potential competitor for *Arabidopsis,* therefore the adaptive plastic responses we observed can be considered an example of novel interactions mediated though plasticity. The establishment and persistence of plants in novel environments can benefit from plastically generated phenotypic shifts, at times promoting coexistence and enhancing diversity^85–87^.

The third condition for plasticity to evolve is the presence of environmental heterogeneity, which leads to different phenotypes being favored over time and/or space^31^. Based on the variation in reaction norms present across our genotypes, and the inherently variable nature of ruderal habitats over time and space, we can thus infer that under natural conditions the potential exists for variable selection on contrasting phenotypes in *Arabidopsis* that depends on the local competitive conditions*.* For this species and other ruderal plants that occur in temporally dynamic habitats and thus experience a high degree of unpredictability across generations, some plastic responses are expected to be selectively favored over canalized responses^80^, as we have documented.

Despite the factors we have outlined that make the evolution of plasticity likely, harsh growing conditions provide an important constraint on selection for plasticity. Selection on phenotypic plasticity should be reduced in extremely resource-limited environments compared to more benign conditions because of the relative rarity of extreme conditions and the low absolute fitness potential there^88^. Thus, populations in extreme environments may rely more on evolution through genetic changes than adaptive plasticity^88^. In nature, *Arabidopsis* can often effectively minimize interspecific competition by (1) flowering early in the season and/or (2) overwintering as a rosette which provides a competitive advantage by allowing the plant an early growing-season switch from vegetative to reproductive growth^89^. Our experimental design likely represented a high degree of interspecific competition relative to what these genotypes would experience in nature. The density of neighbors was high (12 *L. multiflorum* individuals grown within ~ 7 cm of a focal *Arabidopsis* plant), and both species germinated at the same time, leading to strong interspecific competition from the earliest stages of *Arabidopsis*’ life cycle. Our results indicating significant selection on plasticity thus occur even despite likely constraints on its selection due to the extreme growing conditions we imposed.

### Selection and variable phenotypic correlation matrices

Natural selection is also often constrained by among-trait correlations^24^, thus quantifying a large number of potentially adaptive traits is useful for understanding how multivariate selection compares across different environments^90^. Recent studies have begun to highlight a variety of conditions that influence the degree to which trait matrices overall can shift, as well as the implications of such environment-dependent matrices^28,29,91,92^. One common observation is that plants respond to limiting conditions by increasing phenotypic integration, or the extent to which those traits are correlated^27,93,94^. However, we were surprised to find a greater number of significant trait correlations, and thus greater phenotypic integration, under competition-absent versus competition-present conditions. We hypothesize that this pattern reflects the extremely stressful conditions in our competition-present treatment. Perhaps phenotypic integration increases with greater resource limitation as commonly observed, but only up to some maximum value. Statistically, this could simply reflect an extreme reduction in trait variation among individuals in highly stressful conditions. But, such an observation could alternatively represent biologically-based mechanisms by which extremely harsh conditions fundamentally alter the nature of certain trait correlations. Testing the hypothesis that phenotypic integration is maximized at some intermediate level of environmental stress will require comparisons of multi-trait correlation matrices across manipulated gradients of limiting resources. Given the wide-ranging potential for high magnitudes of abiotic stress in response to climate change, such investigations may prove fruitful.

## Methods

### Study system

*Arabidopsis,* otherwise known as mouse-ear cress or thale cress, is a weedy, primarily self-fertilizing mustard that occupies a wide range of environmental conditions in temperate zones across all continents except Antarctica^95^. As a ruderal species adapted to disturbed conditions, it is also highly sensitive to interspecific competition, showing a reduction in size, flowering time and seed production^96^. *Arabidopsis* is used as a model system in molecular biology and ecology, in part due to its short generation time and wide degree of genetic and phenotypic variation across populations. Because of its use as a model, *Arabidopsis* seeds from many natural populations across the world have been collected and are maintained by stock centers for use in the greater research community. Therefore, from both a logistic and an ecological perspective, this system is well-suited for addressing broad questions surrounding the relationship between competition and selection on phenotypic traits.

In this paper, we present data from an experiment with 35 accessions of *Arabidopsis*, selected from natural accessions maintained by the Arabidopsis Biological Resource Center (ABRC; http://abrc.osu.edu/); an additional ten accessions were initially included in the experiment but are excluded here due to poor survival (see Table S1 for details about all 45 accessions). Accessions were originally chosen to be used in a three-generation field experiment designed to assess the relative importance of propagule pressure and population genetic diversity for colonization success^97^. Thus, the accessions met the following two criteria for that study. First, that they had previously been genotyped by the Borevitz 149 SNP project and genetic markers were available to allow genotype assignment in the field^98^, and second, that the accession was available as a single-seed descent line. We also selected no more than one accession from a given source population (e.g. Col). Based on a large-scale study on the population genetic structure of *A. thaliana* that included these accessions^98^, the selected accessions represent genetically distinct populations. We used seeds that had been sent directly from the ABRC, rather than growing all accessions together for a generation or more to negate potential maternal effects, expecting that maternal effects would be minor for multiple accessions that had all been propagated at the same facility. We recognize that by not bulking seeds ourselves in a common environment, we could be introducing environmental noise to our study due to maternal effects^99–101^. Because *Arabidopsis* is highly selfing and most of its genetic and phenotypic variation is partitioned among populations^19^, we consider individual single-seed descent lines as unique genotypes and refer to them as such throughout this paper.

On 5 June 2012, we planted three seeds of a single genotype in the center of each square pot (9.5 cm L × 9.5 cm W × 8.25 cm H) filled with Metro-Mix (SunGro Horticulture, Agawam, MA). In order to implement an interspecific competition treatment, at the same time we also sowed annual rye (*L. multiflorum*; Pennington, Madison, GA) in half of the pots. Similar to *Arabidopsis*, *L. multiflorum* is a ruderal species naturally distributed in temperate climates and native to Europe, Northern Africa and Asia^102,103^. We chose *L. multiflorum* as a competitor because of its fast growth and because its morphology would result in substantial competition both above and belowground for *Arabidopsis*. Both *Arabidopsis* and *L. multiflorum* occur broadly in Mediterranean sites across Eurasia, making them potential natural competitors. *L. multiflorum* is an effective competitor of *Arabidopi*s^104^, and its perennial relative, *L. perenne* has been used successfully as a competitor in previous *Arabidopsis* studies^20,105^. We recognize that results from our experimental design do not necessarily reveal patterns of natural selection exactly as these species would have experienced them in nature; instead, they represent a model of outcomes due to plant competition in a more general sense, consistent with our study goals.

We added multiple grass seeds to each corner of the competition pots, eventually thinning them to 12 individuals per pot (three per corner). We were aiming for an intense competitive environment relative to previous experiments^19,106,107^. After seeding, all pots were covered with aluminum foil, stratified in a 4 °C cold room for six days to break seed dormancy, and moved to a greenhouse. We randomly assigned the original 45 genotypes and two competition treatments to locations within each of six spatial blocks (one replicate per block), yielding a total of 540 pots.

Temperatures in the greenhouse were allowed to fluctuate between 15 and 25 °C, and no additional lighting was provided. Pots were misted daily for 10 days to keep the soil surface moist during the simultaneous germination of *Arabidopsis* and *L. multiflorum*, after which they were primarily bottom-watered. We surveyed pots daily for emerged seedlings and recorded trait data from only the first *Arabidopsis* seedling to emerge; all subsequent seedlings were removed. Plants did not experience vernalization prior to flowering. Plants were harvested as they senesced naturally, and the experiment was concluded after five months (14 November 2012), once *Arabidopsis* senescence was complete.

### Phenotypic traits

The traits we recorded fell into one of three trait categories, which we distinguish as phenology, growth and architecture. To assess phenology, we recorded *flowering time* as the number of days after the stratification period until flowering initiated (when white petals were first visible); *flowering duration* (the number of days between flower initiation and when flowers were no longer present); and the *total number of rosette leaves* at bolting (a useful indicator of developmental stage at flowering initiation for *Arabidopsis*). We assessed plant growth traits by recording *specific leaf area* from a single leaf collected when flowering began (SLA; cm^2^ g^−1^); *aboveground biomass* (weight of the plant material above ground); and the *length of the longest rosette leaf* as an indicator of plant size. In *Arabidopsis*, the diameter of the rosette (i.e., roughly twice the length of a single rosette leaf) is positively correlated with fresh weight (e.g., r^2^ = 0.99 in Leister et al*.*^85^) and is frequently used as a proxy of overall plant size^86,108^. To assess plant architecture, we recorded the *number of basal branches* (flowering stems coming from the base of the plant) and the *number of apical branches* (as the number of primary flowering branches coming from the first flowering stem). For both of these architectural traits, all plants that survived to flower were considered to have at least one basal and one apical branch. As additional architecture traits we also measured *early internode length* (average of the three internode distances at the basal end of the main stem), as an indicator of the degree of stem elongation at the beginning of flowering, and *late internode length* (average of the three internode distances at the distal end of the stem), which indicates stem elongation later in the life cycle of the plant. Lastly, we assessed fitness at the end of the experiment by counting the *number of fruits* produced per plant. Fruit number is broadly used as a measure of reproductive performance in *Arabidopsis*^19,67,109^.

### Phenotypic plasticity

We calculated phenotypic plasticity (PP) for a given trait (X) in response to competition, combining the phenotypic response of that trait under competition-absent and competition-present conditions. We used the following metric^19,74^: PP = (X_competition absent _− X_competition present_)/X_competition absent_. Phenotypic plasticity is therefore defined by the difference of the trait value under competition-absent minus competition-present conditions, standardized by the trait value under competition-absent conditions. Plasticity values were calculated accounting for the paired design within each block. Thus, for each genotype, we calculated six replicate values of plasticity for all traits by pairing individuals in competition-present and competition-absent conditions within a given block.

For plants missing data for any trait (with the exception of fruit number) we used the mean value of that trait from plants of the same genotype and exposed to the same treatment to calculate plasticity. This procedure was done in only four cases. In instances where *Arabidopsis* died prior to flowering (32 cases out the 420 plants in the 35-genotype dataset presented here), we removed that plant and its corresponding pair from all analyses; in all cases mortality occurred early enough in the experiment that no trait data had been recorded.

### Statistical analysis

To confirm the existence of differential plasticity among genotypes, a requirement for testing the hypotheses in our study, we used mixed model ANOVA. These models included competition treatment and genotype as predictors and traits as response variables to test for: (1) genotype-specific responses to competition in the traits we measured (a genotype × treatment interaction), which would indicate variation in trait plasticity among genotypes; (2) significant trait variation among genotypes, indicating genetic differentiation in trait means independent of the environment; and (3) significant variation by treatment, indicating significant trait plasticity independent of genotype identity. Block was included as a random effect. Measured variables deviating from normality or homoscedasticity were transformed using log transformation^110^; this applied to all traits except flowering duration, number of rosette leaves and basal branches. For these analyses, we had between two and six replicate pairs per genotype from both competition treatments for a total sample size of 356 plants across 35 *Arabidopsis* accessions. To assess whether particular genotypes consistently outperformed the others, genotype-specific trait and trait plasticity values within each competitive environment were ranked and compared.

#### Genotypic selection on traits

To explore the role of selection on individual traits, we used multiple regression of genotype mean values to connect our phenology, growth, and architectural traits with relative fitness^24^. In these analyses all traits were standardized and all fitness estimates relativized within competition treatments^19,46,76^. Thus, genotypic selection analysis (GSA) was conducted in each competition treatment separately (n = 35 genotypes in competition-absent and 35 in competition-present conditions). A preliminary GSA with data from both competition treatments combined indicated significant trait × treatment interactions for four out of ten traits (aboveground biomass, longest rosette leaf length, basal branches and apical branches; data not shown), indicating that direct selection varied based on the competitive environment and justifying separate analyses by treatment. Total selection on each trait was estimated by calculating selection differentials (S), the covariance between relative fitness and standardized trait values; we inferred significance based on *P* values from correlations. Direct selection was estimated using multiple regression, with all nine of our focal traits plus aboveground biomass as predictors and relative fitness as the response. We included aboveground biomass as a covariate to account for scaling relationships^46^, but we do not report or interpret significant regression coefficients for biomass. We excluded basal branches from the analysis for the competition-present conditions due to lack of variation among genotypes. We interpret the regression coefficients for all traits as linear selection gradients (*β*). We also estimated quadratic selection gradients (γ) for each trait to infer if nonlinear selection was occurring in our system. Consistent with standard practices, we estimated quadratic selection gradients using a separate model that included all traits as both main effects and polynomial terms, interpreting only the polynomial terms^24^. Reported quadratic regression coefficients were doubled in order to avoid underestimation of the strength of nonlinear selection^76^. Inferences regarding significance of linear and nonlinear selection gradients are based on bootstrapped 95% confidence intervals.

#### Genotypic selection on plasticity

We carried out one additional set of genotypic selection analyses^24^ to test for the relationship between trait plasticity and fitness. Within each treatment, we conducted GSA using a “simple” and a “full” model as has been used previously to make inferences about selection on plasticity^19,74^:

$${\text{W}} = {\text{X}} + {\text{PP}}\_{\text{X}}$$

In the simple model, relative fitness (W) in a given competitive treatment was regressed against the value of a single trait value (X) in that treatment and that trait’s plasticity (PP_X), including aboveground biomass and its plasticity as covariates. In the full model the plasticity and trait value for all nine focal traits plus aboveground biomass were included, better accounting for the covariance structure among all traits and trait plasticities. In all models, significant negative regression coefficients for trait plasticity were interpreted as maladaptive plasticity and significant positive coefficients were interpreted as adaptive plasticity^19^.

Reporting results from both the simple and full models allows us to account for the potential effects of traits correlated with plasticity on relative fitness. Inferences from the literature about selection on plasticity typically use models similar to our simple model^19,38,62,77,111^, but ignoring correlated traits may generate misleading inferences regarding the role of selection on plasticity. Trait correlations vary across different environments therefore the covariance with trait plasticity can as well^112,113^. Also similar to other studies, our inferences about selection on plasticity are based on separate models for competition-present and competition-absent conditions. We used this approach in part because environment specific differences in most trait values (and their variances) from our study were so stark. Additionally, the use of environment-specific models enables us to make inferences regarding selection magnitudes that reflect the trait variation expressed and therefore available for selection to act upon in those specific conditions.

#### Environment-dependent changes in trait correlations

We constructed treatment specific correlation matrices among all traits and among traits and trait plasticity. To visualize the overall structure of correlations among traits, and to assess how the competitive environment may influence the trait covariance matrix, we performed a principal component analysis on centered and standardized trait values from each competition treatment separately.

## Supplementary information

Supplementary Tables.

Supplementary Legends.
