# Supplementary material for: Natural selection on traits and trait plasticity in Arabidopsis thaliana varies across competitive environments
Source: Sci Rep. 2020 Dec 10;10:21632. doi: 10.1038/s41598-020-77444-w (PMC7728774; doi:10.1038/s41598-020-77444-w)
Supplement: Supplementary file 1 — Supplementary Tables. [file 41598_2020_77444_MOESM1_ESM.pdf]

Supplementary Information

Natural selection on traits and trait plasticity in *Arabidopsis thaliana* varies across competitive environments

Kattia Palacio-Lopez, Christian M. King, Jonathan Bloomberg, and Stephen M. Hovick

Table S1

	Stock number	Country	Abbreviation
	CS6740	Austria	In-0
	CS6603	Belgium	An-1
§	CS6626	Czech Republic	Br-0
§	CS6086	England	PHW-30
§	CS6702	France	Et-0
	CS6784	France	Lm-2
	CS6788	France	Lz-0
	CS6100	Germany	Kelsterbach-1
	CS6602	Germany	Ak-1
	CS6612	Germany	Bd-0
	CS6613	Germany	Be-0
	CS6631	Germany	Bsch-2
	CS6679	Germany	Db-2
✠	CS6691	Germany	Ei-5
	CS6693	Germany	Eil-0
	CS6695	Germany	En-1
§	CS6698	Germany	Er-0
	CS6712	Germany	Fr-6
	CS6722	Germany	Gö-2
	CS6757	Germany	Kl-1
	CS6800	Germany	Mz-0
	CS6804	Germany	Nie-0
	CS6821	Germany	Old-2
	CS6831	Germany	Pf-0
	CS6843	Germany	Pt-0
	CS6883	Germany	Vi-0
	CS6643	Ireland	Bur-0
	CS6674	Italy	Ct-1
	CS6825	Italy	Pa-1
	CS6799	Libya	Mt-0
	CS6824	Norway	Oy-0
	CS6669	Portugal	Co-1
§	CS6797	Russia	Ms-0
	CS6828	Russia	Per-1
	CS6865	Russia	Stw-0
§	CS6688	Scotland	Edi-0
	CS6754	Scotland	Kil-0
	CS6769	Scotland	Lc-0
§	CS6621	Spain	Bla-6
	CS6852	Spain	Se-0
§	CS6873	Spain	Ts-7
	CS6178	Tadjikistan	Hodja-Obi-Garm
	CS6188	United States	Seattle-0
§	CS6877	United States	Tul-0
	CS907	United States	Col-2

Table S2

	Aboveground biomass	Flowering time	Flowering duration	Number of rosette leaves	Long. rosette leaf length	Specific leaf area	Basal branches	Apical branches	Early int. length	Late int. length	PP Abovegrd. biomass	PP Flowering time	PP Flowering duration	PP Num.of rosette leaves	PP rosette lf. length	PP Specific leaf area	PP Basal branches	PP Apical branches	PP Early int. length	PP Late int. length
**Aboveground biomass**		0.033	0.533	0.584	0.796	-0.067	0.129	0.406	0.563	0.232	-0.225	0.176	-0.395	-0.106	-0.095	-0.016	0.450	0.063	-0.250	*-0.283*
**Flowering time**	0.737		-0.078	0.571	-0.101	-0.612	0.444	0.128	-0.465	-0.123	0.413	-0.740	*0.295*	0.762	0.663	-0.347	-0.037	*0.297*	-0.270	0.172
**Flowering duration**	0.738	0.642		0.490	0.551	-0.176	-0.080	0.512	0.337	0.152	-0.264	0.148	-0.879	-0.226	-0.139	0.275	0.216	-0.271	0.056	*-0.330*
**Number of rosette leaves**	0.784	0.948	0.538		0.572	-0.591	0.199	0.451	-0.057	0.119	0.062	*-0.310*	-0.365	0.347	0.382	-0.135	0.037	0.003	-0.196	*-0.291*
**Longest rosette leaf length**	0.815	0.778	0.464	0.856		-0.104	0.020	*0.297*	0.522	0.256	-0.180	0.246	-0.473	-0.193	-0.188	0.025	0.282	0.035	-0.181	-0.352
**Specific leaf area**	-0.551	-0.769	-0.458	-0.820	-0.722		-0.166	-0.211	*0.299*	-0.012	-0.263	0.587	0.031	-0.415	-0.430	-0.245	0.123	-0.016	-0.003	0.051
**Basal branches**	0.721	*0.327*	0.645	*0.297*	0.422	-0.046		0.500	-0.198	-0.067	*0.283*	0.054	0.344	0.626	0.376	-0.612	0.244	0.062	-0.835	*0.315*
**Apical branches**	0.801	0.773	0.582	0.795	0.699	-0.504	0.405		0.076	0.123	-0.088	0.114	*-0.327*	0.257	0.234	-0.248	0.156	-0.505	-0.464	-0.120
**Early internode length**	*-0.303*	-0.654	-0.487	-0.557	-0.260	0.514	0.007	-0.369		0.462	-0.404	0.405	*-0.312*	-0.544	-0.551	0.240	0.379	-0.019	-0.162	-0.335
**Late internode length**	0.399	0.496	0.698	0.348	0.151	-0.229	*0.296*	0.357	-0.599		0.009	0.226	-0.131	-0.056	0.082	-0.096	0.052	0.108	-0.058	-0.695
**PP Abovegrd. biomass**	0.549	0.343	0.439	0.347	0.494	-0.344	0.390	0.397	-0.153	0.140		-0.366	0.436	0.472	0.632	-0.203	0.276	0.475	-0.059	-0.077
**PP Flowering time**	-0.208	-0.278	-0.106	-0.385	-0.253	0.483	0.154	*-0.293*	0.199	0.017	-0.366		-0.238	*-0.331*	-0.416	-0.067	0.101	-0.159	-0.182	-0.129
**PP Flowering duration**	0.242	0.266	0.526	0.134	0.087	-0.227	0.174	*0.283*	-0.448	0.504	0.436	-0.238		0.390	0.263	-0.386	0.001	0.387	*-0.300*	0.409
**PP Num. of rosette leaves**	0.602	0.871	0.515	0.830	0.728	-0.726	0.205	0.624	-0.580	0.391	0.472	*-0.331*	0.390		0.837	-0.539	-0.095	*0.294*	-0.396	0.105
**PP Long. rosette lf. length**	0.559	0.670	*0.326*	0.691	0.780	-0.662	0.233	0.495	*-0.295*	0.135	0.632	-0.416	0.263	0.837		-0.382	0.022	0.258	-0.080	-0.154
**PP Specific leaf area**	-0.479	-0.589	-0.486	-0.495	-0.479	0.527	-0.237	-0.387	0.554	*-0.332*	-0.203	-0.067	-0.386	-0.539	-0.382		0.004	-0.151	0.588	-0.041
**PP Basal branches**	0.524	0.023	0.366	0.052	0.258	0.197	0.869	0.268	*0.294*	0.046	0.276	0.101	0.001	-0.095	0.022	0.004		0.141	-0.190	-0.085
**PP Apical branches**	0.380	0.259	0.274	0.235	*0.332*	-0.105	0.172	0.604	-0.058	0.048	0.475	-0.159	0.387	*0.294*	0.258	-0.151	0.141		-0.042	-0.098
**PP Early int. length**	-0.574	-0.573	-0.719	-0.487	-0.359	0.339	-0.467	-0.409	0.619	-0.634	-0.059	-0.182	*-0.300*	-0.396	-0.080	0.588	-0.190	-0.042		-0.267
**PP Late int. length**	-0.085	0.151	*0.333*	-0.003	*-0.295*	0.048	0.000	0.008	-0.572	0.674	-0.077	-0.129	0.409	0.105	-0.154	-0.041	-0.085	-0.098	-0.267	

P < 0.001	0.001 < P < 0.01	0.01 < P < 0.05

Table S3

	Competition absent				Competition present			
	**Axis 1**	**Axis 2**	**Axis 3**	**Axis 4**	**Axis 1**	**Axis 2**	**Axis 3**	**Axis 4**
Aboveground biomass	0.897	0.318	0.206	0.013	0.853	0.236	0.036	0.063
Phenology traits								
Flowering time	0.939	-0.221	-0.015	0.057	0.206	-0.855	-0.143	-0.199
Flowering duration	0.789	0.364	-0.34	-0.182	0.735	0.176	-0.08	0.36
Number of rosette leaves	0.927	-0.264	0.182	0.057	0.8	-0.433	-0.271	0.032
Growth traits								
Longest rosette leaf length	0.831	-0.065	0.46	-0.089	0.819	0.301	-0.113	0.101
Specific leaf area	-0.754	0.483	-0.148	0.347	-0.367	0.667	0.42	0.133
Architectural traits								
Basal branches	0.518	0.796	0.094	-0.143	0.236	-0.526	0.724	-0.189
Apical branches	0.836	0.054	0.168	0.475	0.644	-0.208	0.548	0.091
Early internode lengths	-0.598	0.413	0.577	-0.011	0.422	0.772	0.052	-0.189
Late internode lengths	0.554	0.159	-0.74	0.057	0.338	0.359	-0.073	-0.803

Table S4

Accessions	Fruit number			Above ground biomass			Flowering time			Flowering duration			Number rosette leaves		
	no-comp	comp	pp	no-comp	comp	pp	no-comp	comp	pp	no-comp	comp	pp	no-comp	comp	pp
CS6788	8	**1**	27	9	**1**	24	28.5	26	19	20	**1**	34	5	**1**	27
CS6843	35	2	35	34	11	34	28.5	28	26	13	3	30	6.5	4	13
CS6821	4	3	17	4	2	13	24	20	8	25.5	2	35	12	6	19
CS6669	19	4.5	31	25	14	28	15	10	6	28	6	32	15	8	24
CS6831	11	4.5	22	10	8	22	13	15	13	25.5	7	31	13	5	29
CS6100	22	6	29	23	9	32	15	13.5	10	32.5	32	12	20	17.5	23
CS6722	28	7.5	32	28	12	30	4	6	12	32.5	10	28	21.5	26.5	17.5
CS6693	14	7.5	23	17	27	15	7	8	14	29	5	33	26.5	23.5	30
CS6769	29.5	9	33	24	16	26	11	7	5	14	9	23	30	20.5	33
CS6800	32	10.5	34	29	4	35	21	5	3	23	8	29	23	25	28
CS6799	27	10.5	30	33	24	29	18	**1.5**	4	24	21	19	29	20.5	32
CS6674	21	12.5	28	27	22	25	3	**1.5**	16	17	13	21	33	32.5	21
CS6631	9	12.5	19	5	3	16	6	16	23	3	16	5	31	23.5	35
CS6883	16	14	24	30	15	33	**1**	3.5	20	31	25	18	34	30	34
CS6784	10	15.5	16	19	19	20	5	9	18	10	30	6	32	31	26
CS6695	7	15.5	12	15	18	19	25.5	3.5	**1**	5	18.5	14	26.5	32.5	10
CS6679	2	17	9	7	13	5	12	12	11	18	15	20	18.5	17.5	22
CS6865	3	18.5	7	8	17	7	15	27	30	4	22.5	8	14	11	14
CS6825	6	18.5	11	11	32	4	9	17	21	8.5	4	27	24.5	28	25
CS6757	5	20	6	6	7	9	22	24	24	21	12	24	9	8	11
CS6824	23	21	21	14	30	8	19	25	28	27	31	10	18.5	20.5	20
CS6754	24	22	20	13	20	18	8	18	25	19	27	13	24.5	29	15
CS6602	33	24	26	32	25	27	10	20	22	35	33	16	21.5	26.5	17.5
CS6178	20	24	15	16	29	11	31	31	29	11	18.5	17	6.5	12	5
CS6804	18	24	14	20	31	10	30	22	9	22	28	15	10	15	6
CS6612	34	26.5	25	35	26	31	20	20	17	34	20	25	17	13.5	16
CS6740	**1**	26.5	3	3	5	3	33	34	34	6	24	9	2	2.5	4
CS6712	31	28	18	26	35	12	23	30	31	7	34	3	16	20.5	12
CS6613	25	29	13	22	21	23	27	23	15	30	14	26	8	8	8
CS6852	12	31	4	**1**	6	2	35	35	32	2	17	4	**1**	2.5	**1**
CS6828	13	31	5	12	23	14	25.5	33	35	15	11	22	11	10	9
CS907	26	31	10	21	28	17	17	11	7	8.5	35	**1**	28	34	7
CS6603	29.5	33	8	31	33	21	2	13.5	27	12	29	7	35	35	31
CS6188	17	34	2	18	34	6	32	32	33	16	26	11	4	16	3
CS6643	15	35	**1**	2	10	**1**	34	29	2	**1**	22.5	2	3	13.5	2

Accessions	Long. rosette leaf length			Specific leaf area			Basal branches			Apical branches			Early internode length			Late internode length		
	no-comp	comp	pp	no-comp	comp	pp	no-comp	comp	pp	no-comp	comp	pp	no-comp	comp	pp	no-comp	comp	pp
CS6788	4	**1**	19	27	27	20	20	19	20	7.5	4	16	18	11	25	31	21	29
CS6843	21	12	23	28	34	12	30.5	19	30.5	28	2	35	25	15	28	16	22	16
CS6821	7	3	12	7	5	24	10.5	19	10.5	6	3	24	4	2	21	32	5	34
CS6669	15	6	22	15	11	23	27.5	19	27.5	15.5	20	12	20	22	12	35	30	28
CS6831	18	7	25	22	21	13	7.5	19	6.5	32	6.5	32	12	13	19	27	24	25
CS6100	11	2	24	16	12	19	27.5	19	27.5	18	29.5	11	16	**1**	31	21	17	21
CS6722	25	22	21	20	26	6	22	19	22	30	6.5	31	14	8	23	8	27	9
CS6693	22	5	29	9	15	10	18	19	18	23	20	23	2	17	7	23	14	27
CS6769	30	18	27	3	8	14	9	19	8	35	11.5	34	22	9	29	17	28	12
CS6800	28	11	32	5	6	21	15.5	19	15.5	17	15	17	9	3	26	13	9	19
CS6799	34	17	35	6	7	22	27.5	19	27.5	21	29.5	15	30	6	33	6	13	11
CS6674	31	14	31	**1**	10	4	13.5	19	13.5	15.5	29.5	9	6	4	22	14	12	18
CS6631	26	4	34	10	22	5	5.5	19	4.5	12	20	10	10	5	24	3	18	5
CS6883	33	26	30	14	4	29	12	19	12	25	29.5	19	11	10	18	15	10	20
CS6784	32	19	33	8	20	3	17	19	17	13	29.5	8	23	14	27	24	32	14
CS6695	19	27	16	2	**1**	27	3.5	19	2	5	29.5	4	15	19	17	20	26	15
CS6679	9	10	11	19	14	18	2	19	**1**	11	29.5	6	7	16	11	30	15	30
CS6865	10	24	6	18	32	**1**	5.5	19	4.5	4	20	7	8	12	13	22	16	22
CS6825	17	31	13	21	25	7	20	19	20	14	6.5	18	19	23	10	12	4	23
CS6757	8	13	7	24	33	2	7.5	19	6.5	9	6.5	14	**1**	18	4	34	2	35
CS6824	27	33	18	13	16	11	15.5	19	15.5	26	29.5	20	26	35	**1**	18	35	**1**
CS6754	23	20	20	11	19	8	13.5	19	13.5	28	20	27	3	7	14	19	6	24
CS6602	20	30	15	4	9	15	24.5	19	24.5	34	11.5	33	27	26	16	4	19	6
CS6178	6	8	8	31	30	25	20	19	20	28	29.5	21	29	32	9	9	25	13
CS6804	5	23	3	32	28	30	30.5	19	30.5	19	11.5	22	5	29	3	26	**1**	33
CS6612	29	21	26	23	17	16	33.5	19	33.5	33	20	30	24	33	2	11	31	7
CS6740	2	9	2	33	18	34	10.5	19	10.5	2	11.5	2	31	20	32	33	11	31
CS6712	24	28	17	26	24	26	33.5	19	33.5	20	29.5	13	32	24	30	10	8	17
CS6613	13	25	14	29	23	28	33.5	19	33.5	24	20	25	28	25	20	28	3	32
CS6852	**1**	16	**1**	35	35	33	3.5	2	9	**1**	11.5	**1**	34	28	34	2	7	4
CS6828	12	29	9	30	31	17	24.5	19	24.5	22	11.5	26	13	30	5	29	33	8
CS907	14	32	10	17	2	32	27.5	19	27.5	10	29.5	5	21	27	8	7	20	10
CS6603	35	34	28	12	3	31	23	19	23	31	16	29	17	21	15	25	23	26
CS6188	16	35	4	25	29	9	33.5	19	33.5	3	29.5	3	33	34	6	5	34	2
CS6643	3	15	5	34	13	35	**1**	**1**	3	7.5	**1**	28	35	31	35	**1**	29	3
