# Supplementary material for: Natural selection on traits and trait plasticity in Arabidopsis thaliana varies across competitive environments
Source: Sci Rep. 2020 Dec 10;10:21632. doi: 10.1038/s41598-020-77444-w (PMC7728774; doi:10.1038/s41598-020-77444-w)
Supplement: Supplementary file 2 — Supplementary Legends. [file 41598_2020_77444_MOESM2_ESM.docx]

**Supplemental material**

Table S1. List of 45 genotypes of *Arabidopsis thaliana* included in the two competition treatments. § indicates genotypes that were excluded because of failure to survive under competition-present conditions. ☩ indicates a single genotype excluded due to failure to reproduce in both treatments.

Table S2. Correlation matrix for traits and trait plasticities in competition-absent (below diagonal) and competition-present (above diagonal) conditions. Significant terms (P<0.05) are shown in bold with increasingly darker shading indicating trait correlations with lower P-values; marginally significant correlations (0.05<P<0.10) are shown in italics. Area within dashed lines correspond to correlation among plasticity only.

Table S3: Principal component loading values for traits of *Arabidopsis thaliana* grown under competition-present and competition-absent conditions.

Table S4: Ranking comparison of phenology, growth, and architectural traits and their plasticities across thirty-five *Arabidopsis* genotypes. For each trait or plasticity value, the putative “best” phenotype is ranked as one, which is shown in bold font against a background of darker shading. Accessions are sorted by the rank of fruit number under competition-present conditions.
